# Special Issue: *Alternaria alternata*: Should We Still Consider This Fungus as a Minor Brother in the Etiology of Respiratory Allergy?

**DOI:** 10.3390/jof8080796

**Published:** 2022-07-29

**Authors:** Jorge Martínez Quesada

**Affiliations:** Department of Immunology, Microbiology and Parasitology, Faculty of Pharmacy, Laboratory of Parasitology and Immunoallergy Lascaray Research Center, University of the Basque Country (UPV-EHU), 01006 Vitoria-Gasteiz, Spain; jorge.martinez@ehu.eus

Allergic diseases represent a major global health problem. The prevalence data of allergic diseases, the high cost of their management and their increasing incidence have sustained the growing interest in the study of this subject. 

Fungi are the third most common cause of type I allergic respiratory diseases, and sensitization to some species of fungi is considered a relevant risk factor for severity in asthma. *Alternaria alternata* is accepted as the most important fungal species causing sensitization, especially in the Mediterranean area. 

Even though fungi are mainly decomposers in ecosystems and play a key role in nutrient cycling, they can also be characterized by biological adaptations that lead to the colonization, infection and/or immunological reactions of other organisms, including plants, animals and humans, causing some kind of damage to them. Pathogenic fungi included in the *Alternaria* genus represent a very limited subset in the huge diversity of organisms belonging to the Fungi kingdom [1].

The scientific publications on *Alternaria* are extensive. However, a number of questions have still not been fully resolved. Among these challenges, we can mention the identification of *Alternaria* species and other related genera; indoor and outdoor aerobiology and their implications in allergy development; allergenic composition and the role of different allergens in diagnosis; cross-reactivity and treatment of IgE-mediated allergy caused by *Alternaria*-related fungi; and the biological role of their allergens in potential pathogenicity or defense phenomena. In the same way, there is a similar requirement to elucidate the aspects of the plant-pathogenicity caused by the different *Alternaria* pathotypes adapted to each plant species [2].

Out of more than 70,000 fungal species described in the literature, Simon Nobbe and cols [3] only refer to somewhat less than 100 genera that have been described as allergenic sources and more than 200 individualized allergens that belong to 24 genera of fungi. Data from Allergome (www.allergome.org, accessed on 10 July 2022), a platform for allergen knowledge, shows 606 allergenic proteins (including isoforms) belonging to 150 fungal species. The WHO/IUIS Allergen Nomenclature Sub-Committee (www.allergen.org, accessed on 10 July 2022) recognizes 120 allergen proteins belonging to 31 fungal species. 

In view of these data, we may ask ourselves whether all sources of fungal allergens are equally relevant in the diagnosis of a fungal allergy. Are these 120 recognized fungal allergens enough for the efficient diagnosis of a fungal allergy? 

Studies on aerobiology, the cutaneous reaction to fungal allergens and allergenic characterization, suggest that the minimum number of species that will ensure an acceptable diagnosis of an allergy to fungi would include the following: *A. alternata, Aspergillus fumigatus, Cladosporium herbarum, Epicoccum nigrum, Fusarium roseum, Penicillium crisogenum, Candida *spp*., Malassezia *spp**. and perhaps some others such as *Helminthosporium* spp., *Trichoderma* spp. and *Aureobasidium* spp. [4].

Postigo and colleagues [5] and Gabriel and colleagues [6] suggest that *A. alternata* and other homologue Pleosporaceae species’ spores are a common aeroallergen source that is found in worldwide environmental samples. Intense exposure to these sources can occur in both outdoor and indoor environments and favors increased levels of exposure to fungal aeroallergens. A consequence of human exposure to *A. alternata* has been unequivocally associated with increased asthma severity.

The major allergen, Alt a 1, has been reported as the main elicitor of airborne allergies in patients affected by a mold allergy in Europe, and can be considered as a marker of primary sensitization to *A. alternata* and homologue Pleosporaceae species. The study and understanding of *A. alternata* allergen information may be the key to explaining why sensitization to *A. alternata* is a risk factor for asthma. Compared to other common environmental allergenic sources, such as pollens and dust mites, fungi are reported to be neglected and underestimated. 

Current research studies on the biological role, sources, identification and characterization of *A. alternata* allergens have allowed for the consideration of new perspectives in the categorization of allergenic molds, assessment of exposure and diagnosis of fungi-induced allergies.

Without doubt, all of that mentioned above justifies the importance of *Alternaria* as the main fungus responsible for the phenomena associated with respiratory allergy in our environment. Pleosporaceae aerobiology studies represent a relevant added value in the deepening of the allergic phenomenon.

On the other hand, the importance of *Alternaria* species and pathotypes as a plant pathogen and contaminants of several fruits before and after harvesting have encouraged the forthcoming research which studies the pathogenic factors in the host–parasite relationship and the involvement of fruit substrates in the fungal allergenic charge of indoor environments [7,8]. 

This Special Issue features two articles of interest to clinicians who manage allergic patients. Lopez Couso and colleagues [9] highlight the importance and efficacy of Alt a 1 in the diagnosis of respiratory allergy due to *Alternaria*, as the most relevant fungal allergenic source, and, Rodriguez and colleagues [10] demonstrate that immunotherapy with Alt a 1 desensitizes treated patients, reducing their symptoms and medication consumption through the elimination of Alt a 1 sensitization.

Vélez del Burgo and colleagues [11] describe, for the first time, a mouse model of asthma induced only by Alt a 1. They demonstrated that Alt a 1 is capable of inducing a lung inflammatory response with an increase in IgE serum levels mimicking the allergic asthma immune-response when it is administered into BALB/c mice. As the authors cite, this model allows the evaluation of the immune-regulatory or immune-tolerant capacity of several molecules that can be used in targeted immunotherapy for fungal allergic asthma.

De Linares and colleagues [12] carried out an aerobiological study where the composition of the airborne fungal flora through spores was studied and compared with the concentration of Alt a 1 in aerobiological samples.

In the context of the plant pathology of the *Alternaria* species, Dauda and colleagues [13] analyze Cytochrome P450s as proteins involved in primary, secondary and xenobiotic metabolisms with wide applications in agriculture as a target for herbicides or fungicides and in the pharmaceutical industry. This study provides relevant data to understand the biology, physiology and toxigenic potentials of P450 in these fungal genera.

Hernández Ramirez and colleagues [14] review the role of the epithelium in cytokine production, TLR-activated alveolar macrophages and innate lymphoid cells in the adaptive response and suggest to deepen the research on the role of *Alternaria* in the induction of IgE-mediated respiratory diseases. 

Sánchez and colleagues [15] review the data supporting Alt a 1 as a phylogenetic marker, as a protein only belonging to Pleosporaceae species and the proposal of this allergen as a key tool for the diagnosis and immunotherapy of fungal allergy in European countries.

Finally, Martins [16] describes the allergic phenomenum caused by fungal species in the veterinary field and puts on the table the tremendous scarcity of data existing in this regard.

We appreciate the contributions of the authors and thank them for sharing their expertise in this Special Issue on “*Alternaria alternata*: Should We Still Consider This Fungus as a Minor Brother in the Etiology of Respiratory Allergy?”

## Data Availability

Not applicable.
